# Awareness and management of chronic disease, insurance status, and health professional shortage areas in the REasons for Geographic And Racial Differences in Stroke (REGARDS): a cross-sectional study

**DOI:** 10.1186/1472-6963-12-208

**Published:** 2012-07-20

**Authors:** Raegan W Durant, Gaurav Parmar, Faisal Shuaib, Anh Le, Todd M Brown, David L Roth, Martha Hovater, Jewell H Halanych, James M Shikany, Ronald J Prineas, Tandaw J Samdarshi, Monika M Safford

**Affiliations:** 1 University of Alabama at Birmingham School of Medicine, 1717 11th Avenue South, Birmingham, AL 35294, USA; 2 Birmingham Veterans Affairs Medical Center, 700 South 19th Street, Birmingham, AL 35233, USA; 3 University of Alabama at Birmingham School of Public Health, 1665 University Blvd, Birmingham, AL, 35294, USA; 4 Wake Forest University School of Medicine, Medical Center Boulevard, Winston-Salem, NC, 27157, USA; 5 University of Mississippi Medical Center, 2500 North State Street, Jackson, MS, 39216, USA

## Abstract

**Background:**

Limited financial and geographic access to primary care can adversely influence chronic disease outcomes. We examined variation in awareness, treatment, and control of hypertension, diabetes, and hyperlipidemia according to both geographic and financial access to care.

**Methods:**

We analyzed data on 17,458 participants in the **RE**asons for **G**eographic **A**nd **R**acial **D**ifferences in **S**troke (**REGARDS**) study with either hypertension, hyperlipidemia, or diabetes and living in either complete Health Professional Shortage Area (HPSA) counties or non-HPSA counties in the U.S. All analyses were stratified by insurance status and adjusted for sociodemographics and health behaviors.

**Results:**

2,261 residents lived in HPSA counties and 15,197 in non-HPSA counties. Among the uninsured, HPSA residents had higher awareness of both hypertension (adjusted OR 2.30, 95% CI 1.08, 4.89) and hyperlipidemia (adjusted OR 1.50, 95% CI 1.01, 2.22) compared to non-HPSA residents. Also among the uninsured, HPSA residents with hypertension had lower blood pressure control (adjusted OR 0.45, 95% CI 0.29, 0.71) compared with non-HPSA residents. Similar differences in awareness and control according to HPSA residence were absent among the insured.

**Conclusions:**

Despite similar or higher awareness of some chronic diseases, uninsured HPSA residents may achieve control of hypertension at lower rates compared to uninsured non-HPSA residents. Federal allocations in HPSAs should target improved quality of care as well as increasing the number of available physicians.

## Background

The diagnosis and treatment of chronic diseases often takes place in primary care settings [1]. Therefore, patient awareness and management of chronic diseases may vary according to geographic access to primary care. However, traditional “rural” vs. “urban” designations, often based primarily on population density, may be inadequate to capture actual differences in geographic access to primary care [2]. Traditional rural and urban designations often do not account for the availability of health care providers which can vary in geographic areas, independently of the population size [2]. In contrast, federally-designated geographic primary care Health Professional Shortage Areas (HPSA), based on both population size and primary care physician availability, may be better markers for limited geographic access to primary care [3].

The Health Resource and Services Administration (HRSA) designates geographic primary care HPSAs based on 3 criteria [4]. First, a HPSA must be a rational area (e.g. a county, neighborhood, or grouping of demographically similar townships or census tracts) for the delivery of primary medical care services. Secondly, a HPSA must have a population to full-time-equivalent primary care physician ratio of at least 3,500:1. Alternately, a qualifying HPSA may have a population to full-time equivalent primary care physician ratio of less than 3,500:1 but greater than 3,000:1 and have unusually high needs for primary care services or insufficient capacity of existing primary care providers. Finally, HPSAs must demonstrate that primary medical care professionals in contiguous areas are overutilized, excessively distant, or inaccessible to the population under consideration. HPSA designation guides the allocation of federal resources to the underserved areas to increase the number of practicing primary care physicians and increase Medicare reimbursement among physicians practicing in these areas. Residence in a HPSA has been previously associated with lower health care utilization and poor health outcomes in limited populations [5-7]. Yet, less is known about whether these physician shortages are associated with the diagnoses and management of common chronic diseases typically encountered in primary care settings in underserved populations residing in these areas [8].

Though geographic primary care HPSAs are based on geographic access to care, inadequate health insurance still potentially looms as an additional barrier to primary care in underserved communities already beset by geographic barriers to care. Individuals living in less densely populated areas are not only more likely to have lower geographic access to primary care physicians, but also less likely to be insured [9]. Furthermore, several studies have demonstrated that lack of insurance often contributes to poorer outcomes in common chronic diseases that are typically managed in primary care settings [10,11]. Despite federal efforts to overcome geographic barriers to access by increasing primary care physician availability, it is unclear whether lack of insurance remains a financial barrier to access even in the presence of more primary care providers in these underserved areas. Due to multiple types of barriers to access to primary care, residents of medically underserved areas may be particularly vulnerable to both underdiagnosis and suboptimal management of chronic diseases [12]. Though increasing physician availability may improve geographic access to primary care, it may not be sufficient to address persistent financial barriers associated with a lack of insurance. Therefore we sought to examine the influence of physician shortages on awareness, treatment, and control of hypertension, diabetes, and hyperlipidemia among persons both with and without health insurance.

## Methods

Baseline data were analyzed from the REasons for Geographic And Racial Differences in Stroke (**REGARDS**) cohort. REGARDS is a national, population-based longitudinal cohort study of 30,183 self-identified blacks and whites aged 45 and older, living in the community, and balanced on black race and sex by design. The sampling scheme and methods for REGARDS have been described in detail previously [13]. Briefly, the study cohort was recruited with an oversampling of both blacks and persons living in either the Stroke Buckle (coastal plains region of North Carolina, South Carolina, and Georgia) or the Stroke Belt (remainder of North Carolina, South Carolina, and Georgia, plus Alabama, Mississippi, Tennessee, Arkansas, and Louisiana). Written informed consent was obtained from each participant. At baseline, participants underwent computer-assisted telephone interviews (CATI) assessing demographic information, medical history, and functional status. Following the CATI, trained research staff conducted an in-home examination that included blood pressure assessments (average of two measurements taken during same baseline in-home visit) and the collection of blood and urine samples. During the in-home visit, self-administered questionnaires were left with participants to gather information on additional demographic and risk factor characteristics. These questionnaires were completed by the participant after the home visit and were returned by self-addressed, prepaid envelopes. Participants are followed by telephone at 6-month intervals for surveillance of cardiovascular events such as stroke and myocardial infarction. The study methods were reviewed and approved by the University of Alabama at Birmingham Institutional Review Board as well as institutional review boards at the collaborating institutions. This study focuses on the baseline data collected from the CATI, self-administered questionnaires and in-home visits.

The 9 outcomes for the study include the baseline awareness, treatment and control among those with hypertension, diabetes or hyperlipidemia upon study entry. Hypertension was defined by self-reported disease by the participant, or if blood pressure was ≥140/90 mmHg, or if the person had diabetes or renal disease (GFR < 60 mL/min/1.73 m^2^) and blood pressure ≥130/80 [14]. Diabetes was defined as self-reported disease, treatment with a diabetes medication on pill-bottle review, or a single fasting glucose ≥126 mg/dL or random glucose ≥200 mg/dL [15]. Hyperlipidemia was defined as self-reported disease, treatment with a lipid medication on pill-bottle review, or low density lipoprotein cholesterol ≥130 mg/dL [16].

Awareness of disease was self-reported in each of the 3 disease groups (e.g. “Has a doctor or other health care professional ever told you that you had diabetes?”). Treatment of disease was assessed by either self-report (“Are you currently taking diabetes pills or insulin?”) or identification of a medication for the target disease (e.g. oral hypoglycemics or insulin for diabetes) on the medication inventory performed during the baseline in-home visit [17,18]. Control for each disease group was defined by disease-specific targets of therapy. For diabetes, control was defined as a fasting blood sugar <140 mg/dL; for hypertension as a BP <140/90 mmHg (or, for diabetes or CKD, <130/80); for hyperlipidemia as LDL cholesterol <130 mg/dL.

The primary independent variables were residence in a federally-designated geographic primary care HPSA and insurance status at baseline. We used county-level HRSA designations to define all the U.S. counties containing REGARDS participants as complete (n = 2,545), partial (n = 10,236), or non-HPSA counties (n = 17,427). Complete HPSA counties are entirely comprised of HPSAs while non-HPSA counties do not include any HPSAs. Partial HPSA counties are comprised of both HPSAs suffering from limited physician availability and non-HPSA areas without physician shortages. Similar to previous studies using county-level HPSA data [6,19-21], residents of partial HPSA counties were excluded from the analyses due to an inability to determine if REGARDS participants in these counties resided in the HPSA or non-HPSA portion of the county. Only the 17,458 REGARDS participants falling into at least one of the 3 defined disease groups and residing in either complete HPSA or non-HPSA counties were included in these analyses. Baseline insurance status (insured vs. uninsured) was based on self-report (“Do you have healthcare coverage such as health insurance, an HMO or a government plan like Medicare or Medicaid?”).

Other baseline independent variables included sociodemographics (race, age, gender, and education). Body mass index, medication adherence, and tobacco and alcohol use, and frequency of exercise were also included in the analyses. The rurality of the county of residence was based on criteria set by the U.S. Census Bureau [22]. We defined rural counties as those that are 25% or less urban; urban as those that are 75% or more urban; and mixed counties as 25%-75% urban. Models for each target disease category (hypertension, diabetes, hyperlipidemia) were also adjusted for the presence of one or both of the other target diseases.

Unadjusted logistic regression models were constructed to examine the crude relationship between HPSA status and each of the 9 chronic disease outcomes (awareness, treatment or control for the 3 conditions). Interaction terms (insurance status*HPSA residence) were included in each model, with 4 of 9 demonstrating a significant interaction (P < 0.10) between insurance status and HPSA residence (other interaction terms such as race*HPSA and age*HPSA were not significant). Therefore, all analyses were stratified by insurance status to examine the relationship between HPSA status and the 9 chronic disease outcomes separately among the insured and uninsured. Multivariable logistic regression models stratified by insurance status were then created with all the covariates included to determine the independent relationship of HPSA status on all of the 9 chronic disease outcomes.

## Results

### Characteristics of study population

Of the 17,458 REGARDS participants included in the analysis, many fell into one or more of the three disease categories with 12,747 with hypertension, 4,887 with diabetes, and 14,065 with hyperlipidemia. Overall, HPSA residents were slightly more likely to be uninsured (9% vs. 6%, p < 0.001) compared to non-HPSA residents. The characteristics of the study population varied according to HPSA and insurance status (Table 1).

**Table 1:** Characteristics of study population

**Sociodemographic and health related behaviors**		Insured*		**Uninsured**		**P-value**^‡^
		**HPSA**^♦^	**Non-HPSA**	**HPSA**	**Non-HPSA**	
		**n = 2060**	**n = 14247**	**n = 201**	**n = 950**	
Age (%)	< 65	49.8	46.7	92.5	91.6	<0.0001
	≥ 65	50.2	53.3	7.5	8.4	
Gender (%)	Female	53.2	54.2	62.7	62.5	<0.0001
	Male	46.8	45.9	37.3	37.5	
Race (%)	Black	35.0	36.6	50.8	59.7	<0.0001
	White	65.0	63.4	49.3	40.3	
Urban Group (%)	Urban	37.3	69.9	39.3	69.0	<0.0001
	Mixed	21.1	11.0	19.4	10.0	
	Rural	41.6	19.1	41.3	21.0	
Education (%)	< High school	17.4	12.4	22.4	20.1	<0.0001
	High school grad	28.8	25.7	41.8	33.9	
	Some college	22.9	27.3	25.4	28.4	
	College grad and above	30.9	34.6	10.5	17.6	
Income (%)	<$25,000	31.4	26.5	59.7	53.2	<0.0001
	$25,000 to $50,000	32.0	31.1	21.4	24.0	
	>$50,000	24.6	30.3	3.5	9.6	
	Refused	12.0	12.2	15.4	13.3	
Medication Adherence^∞^ (%)	Non-Adherent	31.8	34.5	39.3	41.5	<0.0001
	Adherent	68.2	65.5	60.7	58.5	
Tobacco Use (%)	Current	15.5	13.5	27.6	29.6	<0.0001
	Never	46.1	45.2	45.7	38.9	
	Past	38.4	41.3	26.6	31.5	
Current Alcohol (%)	No	56.0	50.53	59.7	56.7	<0.0001
	Yes	44.0	49.5	40.3	43.3	
Body Mass Index (%)	≤25 kg/m^2^	21.5	23.1	23.2	18.7	<0.0001
	25 to 29.9 kg/m^2^	36.2	37.5	30.3	32.0	
	≥30 kg/m^2^	42.3	39.5	46.5	49.4	
Frequency of Exercise (%)	1 to 3 times per week	33.8	35.6	27.0	37.9	0.0198
	4 or more per week	30.8	29.4	38.8	28.6	
	None	35.4	35.0	34.2	33.4	

**Note:** * = Insurance status assessed as whether or not participant has any form of health insurance; ♦ = HPSA: Health Professional Shortage Area; ∞ = Medication adherence: assessed using the validated 4-item Morisky scale.^**‡**^p-value refers to Chi-square comparisons across both insurance and HPSA strata.

### Awareness, treatment and control

Awareness, treatment and control varied among those with hypertension, diabetes, and hyperlipidemia, and according to HPSA and insurance status (Table 2). The crude and adjusted odds of awareness, treatment and control, among HPSA residents compared with non-HPSA residents, also varied according to insurance status (Table 3). Among the uninsured, persons living in HPSA counties had similar odds of being aware of their diabetes compared to non-HPSA residents. However, uninsured HPSA residents had significantly higher odds of awareness of both hypertension and hyperlipidemia compared to uninsured non-HPSA residents (Table 3). In contrast, among the insured, there were no differences in awareness of disease according to HPSA residence for any of the 3 disease groups. Among the uninsured, there also were no differences in the odds of treatment for hypertension, diabetes, or hyperlipidemia among HPSA residents compared to non-HPSA residents (Table 3). Yet, among the insured, there was a higher odds of treatment among only HPSA residents with diabetes. With regard to disease control, among those uninsured HPSA residents with hypertension, there were significantly lower odds of control compared to those uninsured persons living in non-HPSA counties (Table 3).

**Table 2:** Unadjusted prevalence, awareness, treatment and disease control by HPSA residence and insurance status

	**Insured**		**Uninsured**	
	**HPSA n = 2060**	**Non-HPSA n = 14247**	**HPSA n = 201**	**Non-HPSA n = 950**
Hypertension (%)	74.5	72.6	75.1	74.4
Awareness	89.1	90.0	92.7	90.1
Treatment	95.0	95.6	97.7	92.3
Control	55.4	59.2	39.6	55.4
Diabetes (%)	29.7	27.4	36.3	30.5
Awareness	90.2	90.1	87.7	85.2
Treatment	84.4	80.6	82.8	80.6
Control	38.0	41.0	35.9	36.7
Hyperlipidemia (%)	81.3	80.4	83.6	79.6
Awareness	73.1	75.3	67.9	60.5
Treatment	63.6	64.0	57.9	51.2
Control	75.1	78.5	62.1	68.4
Presence of any of 3 target conditions* (%)				
One	36.5	39.0	34.8	37.6
Two	41.6	41.5	35.3	40.3
Three	21.9	19.5	29.9	22.1

*One = having only one of 3 target conditions (hypertension, diabetes, or hyperlipidemia), 2 = having any 2 of 3 target conditions, 3 = have all three target conditions.

**Table 3:** Crude and adjusted odds ratios and 95% confidence intervals for the association between HPSA residence (reference: non-HPSA residence) and awareness, treatment or control of hypertension, diabetes, and hyperlipidemia, stratified by insurance status

	**INSURED**								**UNINSURED**					
		**CRUDE**			**ADJUSTED**^**†**^				**CRUDE**			**ADJUSTED**^**†**^		
		**Odds ratio**	**95% Confidence interval**		**Odds ratio**	**95% Confidence interval**			**Odds ratio**	**95% Confidence interval**		**Odds ratio**	**95% Confidence interval**	
	**n**		**Lower limit**	**Upper limit**		**Lower limit**	**Upper limit**	**n**		**Lower limit**	**Upper limit**		**Lower limit**	**Upper limit**
HYPERTENSION‡ (n = 12736)														
Awareness*	11451	0.91	0.76	1.08	0.88	0.74	1.06	830	1.40	0.72	2.71	2.30	1.08	4.89
Treatment	10916	0.87	0.67	1.13	0.85	0.64	1.12	751	1.86	0.78	4.43	1.95	0.76	4.98
Control	6364	0.86	0.76	0.97	0.92	0.81	1.04	698	0.53	0.36	0.77	0.45	0.29	0.71
DIABETESβ (n = 4884)														
Awareness	4407	0.95	0.71	1.26	0.97	0.70	1.19	350	1.24	0.57	2.67	1.43	0.50	2.63
Treatment	3575	1.30	1.02	1.66	1.31	1.01	1.71	299	1.16	0.57	2.39	1.32	0.55	3.17
Control	1441	0.88	0.72	1.08	0.90	0.72	1.11	243	0.97	0.51	1.81	1.11	0.54	2.28
HYPERLIPIDEMIAλ (n = 14053)														
Awareness	10416	0.89	0.79	1.00	0.89	0.79	1.01	896	1.38	0.97	1.97	1.50	1.01	2.22
Treatment	6599	0.98	0.87	1.11	0.97	0.85	1.11	553	1.31	0.87	1.98	1.18	0.73	1.92
Control	5118	0.83	0.69	0.98	0.84	0.70	1.02	292	0.76	0.43	1.34	0.72	0.37	1.41

*Insurance status: Defined as whether participants have any type of insurance.

HPSA: Health Professional Shortage Area.

^‡^Hypertension was defined as having a blood pressure ≥ 140/90 or (≥ 130/80 or greater if the participant also had diabetes or chronic kidney disease, defined as estimated glomerular filtration rate (eGFR) <60 ml/min/1.73 m^2^), or report of being told by a doctor or nurse they had hypertension.

^β^Diabetes was defined as a fasting blood sugar ≥126 mg/dL, having been told by a doctor or nurse they had diabetes, or treatment with a diabetes medication.

^λ^Hyperlipidemia was defined as cholesterol ≥130 mg/dL, having been told by a doctor or nurse they had high cholesterol, or treatment with a cholesterol medication.

^†^Adjusted for age, race, gender, income, education, alcohol use, tobacco use, exercise, medication adherence, BMI category, urban group, and comorbid chronic condition(hypertension, diabetes, or hyperlipidemia).

(hypertension, diabetes, or hyperlipidemia).

## Discussion

Our results indicate few differences in awareness, treatment and control among both insured and uninsured persons living in HPSA and non-HPSA counties. Despite higher awareness and similar treatment among uninsured HPSA residents compared to uninsured non-HPSA residents with hypertension, the control of hypertension was significantly lower for those uninsured persons living in HPSA counties compared to non-HPSA counties.

Similar to previous studies focused on awareness, treatment, and control of hypertension, hyperlipidemia, and diabetes [12,23-34], our results suggest that rates of awareness and treatment of these chronic conditions are generally higher than corresponding rates of control. National data on hypertension, diabetes, and hyperlipidemia have revealed large proportions of persons, treated for these chronic diseases and still not well-controlled [25,28]. Separate analyses of National Health and Examination Nutrition Examination Survey (NHANES) data revealed awareness, treatment and control rates of 76%, 65%, and 57% among hypertensives and 71%, 51% and 51% among persons with diabetes [28,31]. Compared to hypertension and diabetes, NHANES data reveals lower awareness, treatment and control rates for hyperlipidemia at 35%, 12% and 5% [25]. While these and other data demonstrate that awareness, treatment, and control rates vary among these 3 common chronic diseases, significant proportions of persons with diabetes, hypertension, and hyperlipidemia who receive treatment are not well-controlled [12,23]. Though our results reveal similar trends toward inadequate control of all 3 common chronic diseases, our study differs from others because we were also able to demonstrate some variation in these measures according to geographic access to care and insurance status.

Among the uninsured only, residence in HPSA counties was associated with significantly higher awareness of hypertension and hyperlipidemia and similar awareness of diabetes compared to those living in non-HPSA counties. Our results are similar to a recent study based on the association of HPSA residence with the prevalence, awareness, and control of cardiovascular risk factors in the Multi-Ethnic Study of Atherosclerosis (MESA) cohort [8]. Similar to our data, this study showed fairly equal awareness of diabetes regardless of HPSA status. The investigators found no differences in awareness of hypertension or hyperlipidemia, while we found higher awareness among uninsured persons living in HPSA counties in both these disease groups [8].

Contrary to our results and those of other studies, one might predict that lower geographic access to primary care providers would lead to fewer physician encounters and lower awareness of disease among HPSA residents [5,20]. However, our seemingly paradoxical findings could be explained if the additional human and financial resources dedicated to HPSAs not only increase geographic access to physicians in underserved geographic areas, but also mitigate some of the financial barriers to access faced by uninsured HPSA residents. Because uninsured non-HPSA residents do not live in geographic areas receiving these additional human and financial resources to increase access to care, they may be no more likely, or in the cases of hyperlipidemia or hypertension, even less likely, to be aware of their disease. For example, federally-funded rural health clinic (RHC) programs are located exclusively in rural HPSAs and allow providers to receive higher reimbursements from Medicare and Medicaid [35]. Though RHCs are not obligated to provide care for the uninsured, they often serve as a major safety net providers in these areas [36,37].

Among the insured, the absence of disparities in awareness of disease based on HPSA residence may also be attributable to common financial access to a wider array of health care choices likely available to both insured non-HPSA and insured HPSA residents. Those with insurance typically have higher access to primary care physicians and medical specialists even when controlling for place of residence [38]. Therefore, the increased financial access to care may buffer some geographic barriers to care even among insured HPSA residents.

Despite the relatively higher odds of awareness and similar odds for treatment for hypertension associated with HPSA residence among the uninsured, the odds of control of hypertension were lower for uninsured HPSA residents compared to uninsured non-HPSA residents. Uninsured HPSA residents did not suffer worse control in the diabetes and hyperlipidemia disease groups. Previous studies have demonstrated that hypertension, compared to other cardiovascular diseases, may be particularly vulnerable to variation in control according to access to care [39]. The lower rates of hypertension control among uninsured HPSA residents compared to uninsured non-HPSA residents may also seem paradoxical juxtaposed to similar rates of treatment of hypertension in the two groups.

While resource allocation aimed at increasing geographic access to care among HPSA residents may have positively impacted rates of chronic disease awareness and treatment among those living in underserved geographic areas, similar ameliorative effects do not seem to have been realized with actual blood pressure control. This dichotomy between treatment and blood pressure control may be due to poorer quality of care among uninsured HPSA residents. Though patients are receiving treatment for hypertension, it is unclear whether providers adhere to guidelines in their treatment of uninsured HPSA residents. Adherence to treatment guidelines, such as the first-line use of thiazide diuretics for stage I hypertension (≥140/80 mmHg) and dual antihypertensive therapy for stage II hypertension (≥160/100 mm Mg) [14], could be added to considerations for renewal of HPSA designations to ensure that continued federal funding is allocated toward increasing quality of care as well as provider availability. Current efforts, such as federal monetary incentives [40], to increase use of electronic health records would facilitate more feasible assessments of outpatient quality measures in HPSAs in the future.

In addition to physician-level factors, uninsured HPSA residents may be more complex patients due to more prevalent sociodemographic and clinical barriers to optimal health. Compared to uninsured non-HPSA residents, uninsured HPSA residents more frequently report fair or poor health and higher prevalences of chronic diseases such as arthritis, obesity, and serious psychological distress [41]. Uninsured patients residing in HPSAs are also more likely than uninsured non-HPSA residents to report family incomes < 200% of the federal poverty level, to be unemployed and to have no high school diploma [41]. All of these characteristics, found more frequently among uninsured HPSA residents, may increase the complexity of such patients, potentially making disease control targets even more elusive [42]. Some observers have advocated including sociodemographic and clinical characteristics among HPSA residents as additional considerations to increase funds allocated to HPSAs to account for the care of groups, such as the uninsured, with potentially higher-than-average health care needs [43].

This study has a few limitations. REGARDS is a nationwide community-based cohort, but it may not be representative of the entire U.S. population. The cohort is a relatively older population comprised exclusively of African Americans and whites so results may be less generalizable to younger populations or other races or ethnicities. We used HPSA designations from 2006 though these designations may change depending on population shifts, changes in the physician workforce, or other local factors [20]. Therefore, we were unable to determine how changes in HPSA designations over time may have influenced our findings. Our data are cross-sectional, without the ability to establish causality. We also used county-level HPSA designations and excluded partial-county HPSAs. This conservative approach [6,19-21] ensured that we did not include persons living in portions of partial-county HPSAs that were not underserved. However, we also miss some participants residing in underserved portions of partial-HPSA counties. Though we measured geographic and financial access to primary care using HPSA and insurance status, respectively, we were unable to measure health care utilization to determine, directly, how each factor might impact our outcomes. However, previous studies have demonstrated that both the uninsured and HPSA residents tend to have lower health care utilization across multiple chronic diseases [5]. In addition to the absence of health care utilization data, we were also unable to determine if HPSA residents accessed care within or outside their counties of residence. However, even if participants were able to access care outside the HPSA-designated area, the impact of longer travel distances for outpatient care has been shown to lead to lower health care utilization for multiple medical conditions independent of HPSA status [44-47].

## Conclusions

In summary, uninsured HPSA residents were similarly or more likely to be aware of chronic disease compared to uninsured non-HPSA residents. However, disease control rates for hypertension are lower for uninsured HPSA residents compared to non-HPSA residents who are uninsured. Federal HPSA designations may have made a positive impact on the identification of chronic disease among the most vulnerable HPSA residents, but the management of these chronic conditions has not been optimized to the same extent. Consequently, in addition to increasing the number of primary care physicians, policymakers should consider other strategies both to improve the quality of care and to address the needs of particularly vulnerable HPSA residents, such as the uninsured.

## Competing interests

The author(s) declare that they have no competing interests.

## Authors’ contributions

RD conceived of the study design, interpreted the data analysis, and drafted and revised the manuscript. GP participated in the conception of the manuscript, conducted portions of the data analysis related to assigning HPSA status to REGARDS participants, and assisted in reviewing and revising the manuscript. FS participated in event ascertainment, conducted portions of the data analysis related to assigning HPSA status to REGARDS participants, and assisted in reviewing and revising the manuscript. AL assisted in conducting and interpreting the data analysis and the review and revisions of the manuscript. TB assisted in the conception of the study, interpretation of the data analysis, and review and revisions of the manuscript. DR assisted in the planning and interpretation of the data analysis and the review and revisions of the manuscript. MH assisted in the planning of the data analysis, conducted portions of the data analysis related to predictive probabilities, and reviewed and revised the manuscript. JH assisted in the conception of the study, interpretation of the data analysis, and review and revisions of the manuscript. JS assisted in the conception of the study, interpretation of the data analysis, and review and revisions of the manuscript. RP assisted in the conception of the study, interpretation of the data analysis, and review and revisions of the manuscript. MS assisted in the conception and design of the study, planning and interpretation of the data analysis, and review and revisions of the manuscript. All authors read and approved the final manuscript.

## Pre-publication history

The pre-publication history for this paper can be accessed here:

http://www.biomedcentral.com/1472-6963/12/208/prepub
